# Vertebrate defense against parasites: Interactions between avoidance, resistance, and tolerance

**DOI:** 10.1002/ece3.2645

**Published:** 2016-12-20

**Authors:** Ines Klemme, Anssi Karvonen

**Affiliations:** ^1^ Department of Biological and Environmental ScienceUniversity of JyvaskylaJyvaskylaFinland

**Keywords:** host–parasite interaction, parasite avoidance, resistance, tolerance, trade‐off

## Abstract

Hosts can utilize different types of defense against the effects of parasitism, including avoidance, resistance, and tolerance. Typically, there is tremendous heterogeneity among hosts in these defense mechanisms that may be rooted in the costs associated with defense and lead to trade‐offs with other life‐history traits. Trade‐offs may also exist between the defense mechanisms, but the relationships between avoidance, resistance, and tolerance have rarely been studied. Here, we assessed these three defense traits under common garden conditions in a natural host–parasite system, the trematode eye‐fluke *Diplostomum pseudospathaceum* and its second intermediate fish host. We looked at host individuals originating from four genetically distinct populations of two closely related salmonid species (Atlantic salmon, *Salmo salar* and sea trout, *Salmo trutta trutta*) to estimate the magnitude of variation in these defense traits and the relationships among them. We show species‐specific variation in resistance and tolerance and population‐specific variation in resistance. Further, we demonstrate evidence for a trade‐off between resistance and tolerance. Our results suggest that the variation in host defense can at least partly result from a compromise between different interacting defense traits, the relative importance of which is likely to be shaped by environmental components. Overall, this study emphasizes the importance of considering different components of the host defense system when making predictions on the outcome of host–parasite interactions.

## 1 Introduction

Animals can employ three types of mechanisms as part of their overall defense repertoire to protect themselves from negative effects of parasitic infections (Boots, Best, Miller, & White, 2009; de Roode & Lefevre, 2012). First, they can use mechanisms that prevent or minimize infection (qualitative resistance, see de Roode & Lefevre, 2012), such as behavioral avoidance of habitats, conspecifics, or food that are associated with infections (here termed “avoidance”). The second line of defense consists of mechanisms that reduce infection load or parasite growth after establishment with unspecific and specific immune responses playing a key role (here termed “resistance”). Third, hosts can reduce the negative effects of infection via limitation and repair of damages caused by parasites, that is tolerate infection without preventing the parasite (here termed “tolerance,” e.g., Boots et al., 2009; de Roode & Lefevre, 2012). Despite the benefits of preventing and eliminating infections as well as reducing their negative impact, defense mechanism vary remarkably across individuals, populations, and species (Hart, 2011; Medzhitov, Schneider, & Soares, 2012; Moore, 2002; Råberg, Graham, & Read, 2009; Sadd & Schmid‐Hempel, 2009; Schmid‐Hempel, 2003), suggesting associated costs of defense (Stearns, 1992). Indeed, behavioral parasite avoidance consumes hosts’ time and energy and may result in reduced foraging efficiency or increased exposure to predators (Hart, 1990). Resistance through immune responses incurs deployment costs and can cause immunopathology—a damage of host tissue through immune‐mediated processes (Graham, Allen, & Read, 2005; Lochmiller & Deerenberg, 2000). Further, the maintenance of both immunological defenses and tolerance functions can generate significant costs to the host, leading to reduced fitness in the absence of parasites (Sheldon & Verhulst, 1996; Simms & Triplett, 1994).

Due to these costs, investment into parasite defense can generate trade‐offs with investment into other fitness traits (Sheldon & Verhulst, 1996). Such life‐history trade‐offs are often shaped by environmental and ecological factors that affect host condition and parasite pressure. For example, temperature can affect the probability of infection in a *Daphnia*‐bacteria system, likely through a temperature‐dependent host ability to avoid the parasite (Vale & Little, 2009; Vale, Stjernman, & Little, 2008). Further, levels of immune defense have been shown to vary with environmental conditions, such as resource availability (Siva‐Jothy & Thompson, 2002) or population density (Wilson et al., 2002), as well as parasite species richness experienced by host populations in the past (Corby‐Harris & Promislow, 2008). Also, monarch butterfly populations infected with a protozoan parasite show variation in both resistance and tolerance due to local host–parasite adaptation (Sternberg, Li, Wang, Gowler, & de Roode, 2013). Finally, there is also evidence for tolerance to depend on environmental conditions, such as food and temperature (Vale, Wilson, Best, Boots, & Little, 2011), as well as on the history of exposure to emerging infectious diseases (Adelman, Kirkpatrick, Grodio, & Hawley, 2013). Consequently, optimal defense can show spatial and temporal variation and result in distinct patterns of parasite defense (Lazzaro & Little, 2009; Sandland & Minchella, 2003).

In addition to trade‐offs between distinct life‐history traits, host defense may also exhibit trade‐offs between its branches. However, the relationships between avoidance, resistance, and tolerance have rarely been considered in empirical or theoretical studies (Boots et al., 2009). Recently, a negative relationship between avoidance and tolerance was demonstrated across seven species of tadpoles: shorter lived species, which face comparatively low parasite exposure during their lifetime, invested more into avoidance behavior and less into tolerance, while longer lived species showed the opposite pattern (Sears, Snyder, & Rohr, 2015). A negative relationship has also been demonstrated between resistance and tolerance, both theoretically using epidemiological models (Restif & Koella, 2004) and empirically using inbred mouse strains inoculated with rodent malaria (Råberg, Sim, & Read, 2007). However, there are no empirical studies focusing concurrently on avoidance, resistance, and tolerance in a single host–parasite system.

Knowledge of animal defense mechanisms against parasites, and in particular their interactions, is of fundamental importance as such interactions may have significant implications for host–parasite evolution. For example, both avoidance and resistance negatively affect parasite fitness, resulting in antagonistic coevolution between hosts and parasites, while tolerance is expected to have a neutral or positive effect (reviewed in Råberg et al., 2009). Thus, understanding how hosts balance investment into different defense mechanisms may help predicting the outcomes of parasite–host interactions. Here, we use a natural host–parasite system, the trematode eye‐fluke *Diplostomum pseudospathaceum* and its second intermediate fish host, to study interactions between parasite avoidance, resistance, and tolerance. We observed the three defense mechanisms against *D. pseudospathaceum* individually and also looked at their relationships in two populations of Atlantic salmon, *Salmo salar*, and two populations of sea trout, *Salmo trutta trutta*. We show significant differences in resistance and tolerance between the species, and in resistance between the populations. Further, we demonstrate rare evidence of a trade‐off between resistance and tolerance.

## 2 Methods

### 2.1 Study system

The trematode *D. pseudospathaceum* is an ubiquitous parasite of fresh water systems and has a complex life cycle including three different hosts: aquatic snails, fish, and fish‐eating birds (reviewed in Chappell, Hardie, & Secombes, 1994). In the snail host, the parasite reproduces asexually and produces large amounts of cercariae that actively swim in the water column to encounter fish hosts. As freshwater snails typically occur locally concentrated, infected individuals can create infection hot spots (Jokela & Lively, 1995). After penetration of the fish host, the cercariae migrate to the eye lenses within 24 hr. In the lens, parasites are protected from the host immune system, because this site lacks blood circulation (Chappell et al., 1994). Consequently, our measure of “resistance” (see below) covers mechanisms that act during parasite migration through the host body, after which the parasite cannot be cleared. Subsequently, the parasites develop to metacercariae that become infectious to the definitive host after approximately 5–8 weeks, depending on the temperature. Metacercariae are long‐lived, but do not multiply in the lens (Chappell et al., 1994).

### 2.2 Host and parasite sources

The experiments were conducted in July–August 2014 at Konnevesi Research Station, Central Finland. Two salmonid species, sea trout *S. t. trutta* (hereafter “trout”) and Atlantic salmon *S. salar* (hereafter “salmon”)*,* and two populations of each species were used. The populations originated from different river systems in northern Europe: trout from rivers Ii (Northern Finland, mouth at Gulf of Bothnia: 65°N 25°E) and Ingarskila (Southern Finland, mouth at Gulf of Finland: 60°N, 24°E), and salmon from rivers Neva (Russia, mouth at Gulf of Finland: 59°N 30°E) and Tornio (Northern Finland, mouth at Gulf of Bothnia: 65°N 24°E). One‐year‐old fish were obtained in the end of June 2014 from a fish farm in Central Finland, where they had been raised from eggs received from a breeding program of the Natural Resources Institute Finland. The breeding program aimed at preserving the original genetic background of wild salmonid fish populations and thus, the captive populations are, if possible, regularly supplied with sperm or eggs from the original wild populations.

At the fish farm, all populations were maintained under common garden conditions. Each fish population was held in replicate tanks, which received water from a nearby river inhabited by the snail (*Lymnaea stagnalis*) intermediate host of *D. pseudospathaceum*. Consequently, the incoming water contains parasite cercariae that are haphazardly distributed to the tanks. Parasite exposure in the farm corresponds to natural levels with respect to dose, and the majority of fish of each population harbored infections prior to experimental treatment (Ingarskila: 95.7%, Ii: 88.3%, Neva: 74.5%, Tornio: 60.6%). It is likely that the uninfected individuals had also been exposed and immunized by cercariae that penetrated the fish, but failed to reach the eye lenses.

**Figure 1:**
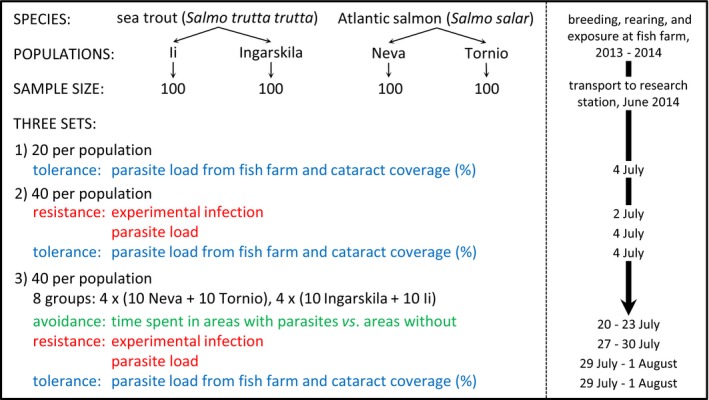
Schematic overview of experimental design

A total of 400 fish, 100 of each population taken from two replicate holding tanks at the farm, were used in the study in three different sets (Figure 1). In set 1, 20 individuals from each population were used to score tolerance and to confirm the developmental stage of parasites obtained at the fish farm. These examinations showed that all infected fish harbored only large fully developed metacercariae. Because metacercariae of different age can be separated according to their size (Sweeting, 1974), it was possible to differentiate infections of an individual that had taken place at the fish farm from those acquired during experimental exposures (see below). In set 2, 40 fish from each population were used for estimations of resistance and tolerance. In set 3, 40 fish from each population were used to determine all three defense traits (see below), totaling 40, 80, and 100 fish from each population scored for avoidance, resistance, and tolerance, respectively (Figure 1).

Parasites used in the experiments originated from 20 *L. stagnalis* snails that were naturally infected with *D. pseudospathaceum*. The snails were collected in July 2014 from Lake Vuojärvi (Central Finland, 62°N, 25°E) and kept at 4°C in individual containers with 1 L of lake water and lettuce ad libitum. It is noteworthy that a study covering a large geographic range in Finland found no evidence of genetic structure in *D. pseudospathaceum* (Louhi, Karvonen, Rellstab, & Jokela, 2010), which is why the origin of the parasites was unlikely to affect the results.

### 2.3 Estimation of avoidance behavior

Forty fish from each population (set 3) were randomly distributed among smaller groups, such that 20 fish of the same species shared one group, with 10 fish from each population (*N *=* *8 groups). The fish species were kept separate to prevent interspecific competition. Within each group, fish were individually marked with visible implant elastomer tags (Northwest Marine Technologies, Shaw Island, Washington) and the maximum number of individual color codes determined the group size of 20. Each group was kept in a 180‐L holding tank and fed daily with commercial fish pellets.

To obtain parasites for the tests, 10 randomly chosen snails were transferred to room temperature and individually placed in 2 dl of lake water (17°C) to stimulate the production of cercariae. After 3 hr, the suspensions from these snails were combined and cercarial density was estimated from ten 1‐ml samples of the mixture.

Behavioral avoidance of parasites was assessed in choice tests measuring the time spent in areas with parasites versus areas without parasites during 20–23 July. The tests were conducted in ten identical tanks (120 × 20 × 20 cm, Fig. S1) with 24 L of lake water (17°C). The tanks were longitudinally divided into three compartments, one smaller middle compartment (28 × 20 × 20 cm) and two larger outer compartments (45 × 20 × 20 cm). A hole with a diameter of 5 cm near the bottom of the separation walls allowed the fish to move through the whole tank. Ten fish from the same group were tested simultaneously. One fish was placed into the middle compartment of each tank and allowed to explore for 1.5 hr to facilitate avoidance behavior (Mikheev, Pasternak, Taskinen, & Valtonen, 2013). All fish visited all compartments of the tank during the habituation. After this period, 150 ml of lake water containing 1,800 parasites (200/L) was added to a randomly chosen outer compartment (P compartment) and 150 ml of lake water without parasites as control to the other outer compartment (C compartment). The solutions were added using a plastic tube that entered just beneath the water surface in the center of each compartment. Pretrials using colored solutions verified that they dispersed throughout the compartment within few seconds, but also stayed well within the compartment. Replicated water samples (*N *=* *3 per compartment in four salmon and four trout) taken after the tests contained an average of 1.33 cercariae/10 ml (range 0.67–2.67) in P compartments, 0.08 cercariae/10 ml (range 0–0.33) in the middle compartments, and no cercariae in the C compartments, indicating that parasites did not spread into the C compartment. Thirty minutes after introducing the parasites, all fish were removed from the experimental tanks and identified. All tanks were subsequently emptied and thoroughly cleaned.

Video cameras placed above the tanks recorded fish behavior. The light conditions were set as dark as the cameras were able to record (15 lux). Recordings were used to quantify the time spent in each of the two outer compartments for 30 min after introduction of parasites. Additionally, swimming activity, that is the time fish spent moving, was estimated during 30 min before and after adding the parasites. The observer was blind to the treatments applied to each compartment.

### 2.4 Estimation of resistance

Resistance was determined by assessing parasite load after exposing fish hosts individually to a controlled number of parasites. First, 40 fish from each population (set 2) were exposed on 2 July by placing them individually in round containers with 2 L of lake water (17°C). Thirteen snails were allowed to produce cercariae for 4 hr, and cercarial density was determined as described above. An estimated total of 400 cercariae were introduced for each fish and the exposure lasted 30 min. Second, all individuals from the experimental groups (set 3) were exposed as described above 7 days after the avoidance tests, that is 27–30 July. The seven‐day interval was used to allow parasite growth so that infections acquired in the avoidance trials and the experimental exposure could be separated according to metacercarial size differences. Parasite load was determined by euthanizing all fish with an overdose of MS‐222 48 hr after exposure, and dissecting their eye lenses.

It is important to note that earlier infections acquired at the farm, whose levels were in the lower end of the variation observed in natural fish populations, were unlikely to affect the measurement of resistance. The number of parasites acquired during the resistance tests was positively associated with farm infection levels. However, this was not due to these farm infections weakening their hosts, as a correlation between the number of parasites acquired at the farm and the condition factor of fish (residual from regression of body length and weight) was not significant (trout: *r*_s_ = −.011, *p *=* *.882, *N *=* *188, salmon: *r*_s_ = .013, *p *=* *.858, *N *=* *193). Instead, the finding suggests that the susceptibility of fish individuals was constant across different environments and patterns of exposure.

### 2.5 Estimation of tolerance

Tolerance is generally defined as host ability to minimize fitness costs of infection at a given parasite load, and fitness costs are commonly assessed indirectly as the degree of pathology caused by the infection (Råberg et al., 2009). Eye‐fluke infections can have serious fitness consequences for fish. Fully developed *D. pseudospathaceum* metacercariae damage the eye lens’ structures and consequently induce cataracts in an intensity‐dependent manner (Karvonen, Seppälä, & Valtonen, 2004a), which impairs host vision (Shariff, Richards, & Sommerville, 1980). Infected fish have been shown to be more prone to predation by birds (final host), and susceptibility to predation increases with cataract coverage (Seppälä, Karvonen, & Valtonen, 2004, 2005). Heavy infections can also lead to impaired growth due to difficulties in visually locating food (Karvonen & Seppälä, 2008). Thus, tolerance was assessed as the area of eye lens covered by cataracts at a given parasite load with more tolerant individuals having smaller areas of cataract coverage at a given load. To estimate cataract coverage, each fish (sets 1, 2, and 3) was examined right after euthanizing using slit‐lamp microscopy (Karvonen et al., 2004a) and the area of lens covered with cataracts was scored as 10%, 20%,…, 100% for each eye. Afterward, both lenses were dissected for parasite load. It is important to note that cataracts are induced mostly by fully developed metacercariae, while new infections less than 12 days old do not induce any cataracts (Seppälä et al., 2005). Thus, in this study, parasite‐inflicted damages were caused by the old infections originating from the fish farm, whereas the few‐days‐old infections acquired during the experimental exposures did not influence tolerance estimation. Further, it should be noted that although resistance was measured based on infections in the laboratory and tolerance based on infections in the farm, both infection environments were similar with respect to water composition and temperature (natural fresh water) and the genetic background of the parasites (Louhi et al., 2010).

### 2.6 Statistics

All statistical analyses were run in SAS v. 9.4 (SAS Institute, Cary, NC, USA). Avoidance behavior was analyzed by fitting a repeated measures generalized linear mixed model (GLMM) with negative binomial error structure. The time spent in the P compartment and in the C compartment (i.e., two values for each individual) was entered as dependent variable and treatment applied to the compartments (P and C) as fixed factor. Additionally, species and population nested within species, as well as their interactions with treatment were entered. Fish ID was included as random repeated factor and fish group as random factor. *p*‐Values in pairwise comparisons of least‐square means were adjusted using the *Bonferroni* correction. The number of parasites obtained at the fish farm did not affect the time spent in the P compartment (GLMM, trout: *F*_1,64_ **= 0.34, *p *=* *.562; salmon: *F*_1,53_ **= 0.01, *p *=* *.917) and was therefore excluded from the final model. Individuals that did not visit one of the outer compartments during the 30‐minute test were excluded (*N *=* *18 salmon and *N *=* *3 trout). Some individuals lost their ID tags and were also excluded (*N *=* *5 salmon and *N *=* *10 trout).

Parasite load acquired during avoidance tests was analyzed using a negative binomial GLMM with load as dependent variable and species as well as population nested within species as fixed factors. There was a strong relationship between parasite load and fish length as well as between parasite load and time spent in the P compartment. However, the slope of these relationships differed between the fish species, and therefore, both variables were included in the model as covariates nested within species. Fish group was included as random factor.

Similarly, parasite load acquired during resistance tests was analyzed by including load as dependent variable and species and population nested within species as fixed factor. Additionally, exposure set (tests conducted in early and late July) and its interaction with species and population, nested within species, were entered as fixed factors. Fish length nested within species was included as covariate.

Analyses on tolerance were conducted in two ways. First, tolerance was quantified on the population level as the slope of a regression of host health (cataract coverage) against parasite load (see Råberg et al., 2009). For this, cataract coverage (average of left and right eye) was entered as response variable into a generalized linear model (GLM) with binomial errors and logit‐link function. Species and population, nested within species, were entered as fixed factors and parasite load (sum of left and right eye) as covariate. Additionally, the quadratic term of parasite load was included to account for a possible nonlinear relationship between cataract coverage and parasite load (Tiffin & Inouye, 2000). To test for differences in tolerance between the fish species and populations, interactions between parasite load and species as well as between parasite load and population, nested within species, were included in the statistical model. As cataracts were never observed in uninfected fish, and all infected fish had at least some cataracts, all uninfected individuals were excluded from the analysis and the intercept was fixed at 0. However, a model including both the uninfected individuals and the intercept yielded a similar outcome (results not shown). Further, for some individuals, it was not possible to estimate parasite load or cataract coverage for both eyes and these were also excluded, resulting in final sample sizes of Ingarskila: *N *=* *90, Ii: *N *=* *82, Neva: *N *=* *71, and Tornio: *N *=* *58.

Second, tolerance was estimated on the individual level as residual deviation from a common regression line between cataract coverage and parasite load across all infected fish individuals. Negative residuals represented relatively small cataracts for a given load (high tolerance) and positive values relatively large cataracts for a given load (low tolerance). Note that our fitness measure cataract coverage was always zero in the absence of infection, which is why we considered this relative measure of damage robust (see Råberg et al. (2009) for discussion on variation in health measures in the absence of infection). Residual cataract coverage was then entered into a GLMM (normal error distribution) with species and population, nested within species, as fixed factors.

Pairwise tests were used to explore the relationship between the three defense traits on the individual and population level. On the individual level, the relationships between avoidance, resistance, and tolerance were analyzed using GLMMs with the proportion of time spent in the P compartment out of the total time spent in the P and C compartment entered as dependent variable (binomial error distribution). Parasite load acquired during the resistance tests nested within population, or the residual cataract coverage nested within population, was entered as factors and fish group as a random factor. Finally, the relationship between resistance and tolerance was analyzed by fitting parasite load acquired during the resistance tests as dependent variable and the residual cataract coverage, nested within population, as factor. On the population level, the relationship between resistance (average parasite load acquired in resistance test, both sets combined) and tolerance (slopes from parasite load vs. cataract coverage regressions) was analyzed using Spearman correlations. However, as this included four populations, we do not report *p*‐values.

## 3 Results

### 3.1 Avoidance behavior

**Figure 2:**
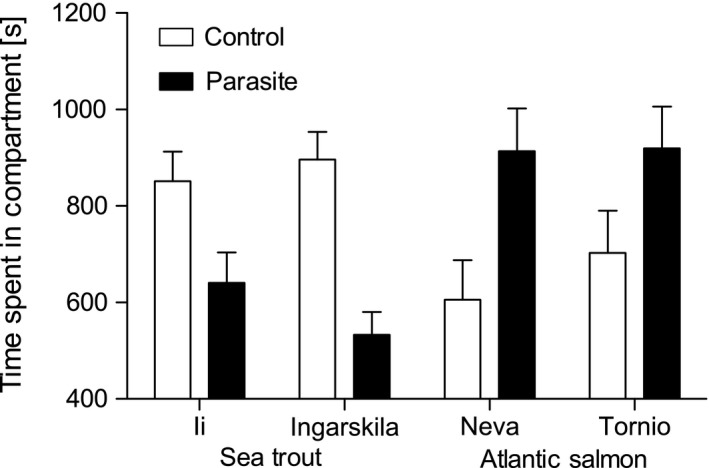
Least‐square mean time ± *SE* spent in control and parasite compartments (in seconds) during a 30‐minute test

The time spent in parasite versus control compartments was affected by an interaction of treatment with species (Table 1). Post hoc pairwise comparisons showed that trout spent significantly less time in the parasite compartment than in the control compartment (*N *=* *67, *t*_240_ = 4.45, *p *<* *.001), while the pattern was opposite in salmon (*N *=* *57, *t*_240_ = −3.47, *p *=* *.001; Figure 2).

**Table 1:** General linear mixed model analyses of time spent in a compartment explained by the treatment applied (parasite vs. control), species, and population

Factors	*df* Denominator	*df* Numerator	*F*	*p*
Treatment	1	240	0.29	.588
Species	1	240	1.93	.166
Population (species)	2	240	0.82	.443
Treatment × species	1	240	42.77	<.001
Treatment × population (species)	2	240	1.54	.216

Fish ID and group are included in the model as random factor to account for dependence of data acquired from the same fish and group.

The position of the fish in the experimental tank at the time of parasite introduction had a significant effect on the time spent in the P compartment for salmon (GLMM, *F*_2,77_ **= 4.37, *p *=* *.016), but not for trout (*F*_2,68_ **= 2.26, *p *=* *.113). When located in the P compartment at the time of parasite introduction, salmon spent significantly more time there (1,196.0 ± 76.3 s), than when located in the C compartment (367.4 ± 77.4 s; *t*_77_ = −2.96, *p *=* *.012). This suggests that salmon developed a preference for one of the compartments during habituation. The distribution of salmon between the compartments was not even at the time of parasite introduction (of 57 individuals, 29 were present in the P compartment and 17 in the C compartment), which may explain the significant preference for the P compartment. Consequently, the avoidance behavior of salmon is not considered in the subsequent trade‐off analyses. Distribution of trout was comparable between P and C compartments at the time of parasite introduction (29 vs. 31). Swimming activity did not differ before and after adding the parasites (*F*_1,244_ **= 0.16, *p *=* *.691), but salmon were generally significantly less active than trout (*F*_1,244_ **= 5.97, *p *=* *.015, interaction not significant.

**Figure 3:**
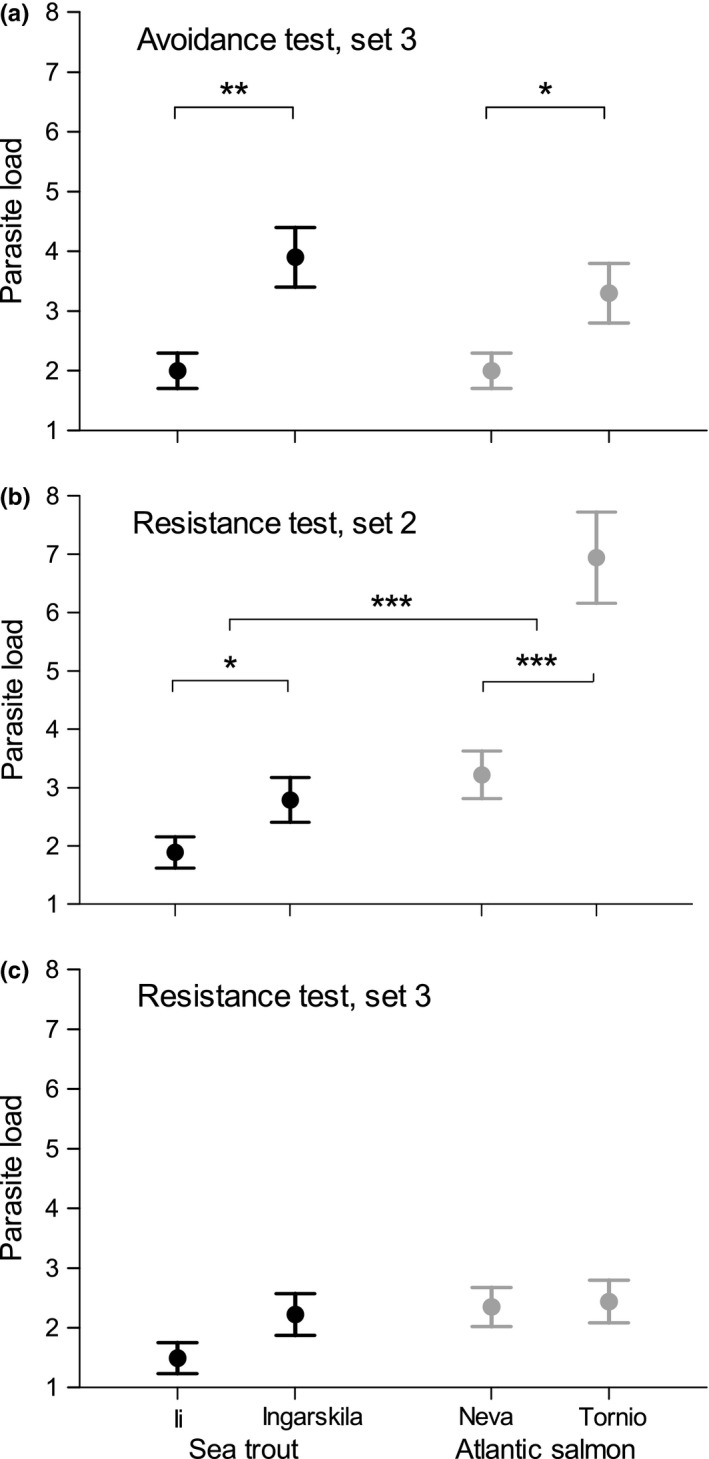
Least‐square means ± *SE* parasite load (number of parasites established in both lenses) acquired during (a) the avoidance test, (b) the resistance test in the beginning of July, and (c) the resistance test in the end of July. Note that individuals used in (a) and (c) are the same, while individuals used in (b) are different. Significant differences are indicated with asterisks (***p <* *.001, **p <* *.010, *p <* *.050)

Parasite load acquired during the avoidance tests did not differ between the species (*F*_1,110_ **= 0.15, *p *=* *.703). Although avoidance behavior was consistent between the two populations of each species (Figure 2), parasite loads were different (*F*_2,112_ = 6.69, *p *=* *.002, Figure 3a), suggesting population‐specific differences in resistance. Post hoc pairwise comparisons showed that trout from Ingarskila had higher parasite loads than trout from Ii (*t*_112_ = −2.84, *p *=* *.005) and salmon from Tornio had higher parasite loads than salmon from Neva (*t*_112_ = −2.31, *p *=* *.023). Further, parasite load increased significantly with the time spent in the P compartment (*F*_2,112_ = 10.76, *p *<* *.001) and decreased with fish length (*F*_2,112_ = 6.01, *p *=* *.003).

### 3.2 Resistance

Parasite load acquired during resistance tests was significantly affected by the interactions between set and species as well as population (Table 2). Post hoc pairwise comparisons indicated that trout showed higher resistance than salmon in set 2 (*N *=* *160, *t*_287_ = 5.42, *p *<* *.001) and the pattern tended to be the same in the third set but was not significant at the five‐percent level (*N *=* *138, *t*_240_ = 1.70, *p *=* *.091, Figure 3b and c). Further, in accordance with parasite load acquired during the avoidance tests, trout from Ii were more resistant than trout from Ingarskila (set 2: *N *=* *80, *t*_*2*87_ = −1.99, *p *=* *.048, set 3: *N *=* *65, *t*_*2*87_ = −1.80, *p *=* *.073) and salmon from Neva were more resistant than salmon from Tornio, but only in set 2 (set 2: *N *=* *80, *t*_287_ = −4.57, *p *<* *.001, set 3: *N *=* *73, *t*_*2*87_ = −0.18, *p *=* *.855). Post hoc comparisons between the sets showed that salmon from Tornio had a higher parasite load in set 2 compared with set 3 (*N *=* *80, *t*_*287*_ = 5.71, *p *<* *.001), while all other populations did not differ (all *p *>* *.098), suggesting increased resistance in fish from Tornio following the avoidance tests. Again, parasite load decreased with length in both species (Table 2).

**Table 2:** General linear mixed model analyses of parasite load acquired during resistance tests explained by species, population, set (tests in early and late July), and length

Factors	*df* Denominator	*df* Numerator	*F*	*p*
Species	1	287	1.50	.222
Population (species)	2	287	8.12	<.001
Set	1	287	21.32	<.001
Species × set	1	287	5.08	.025
Population (species) × set	2	287	3.86	.022
Length (species)	2	287	12.57	<.001

### 3.3 Tolerance

**Figure 4:**
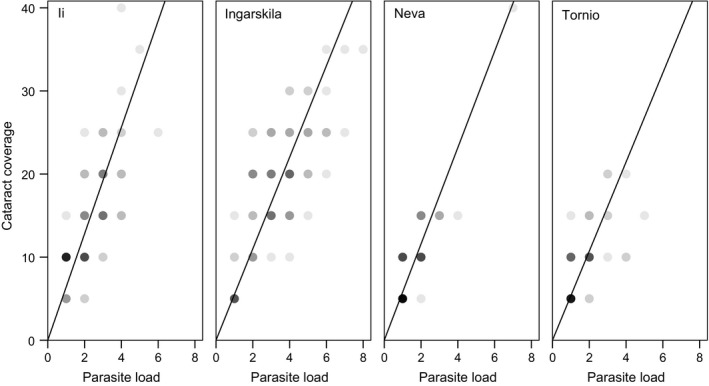
Effect of parasite load (sum of left and right eye) on coverage of parasite‐induced cataracts (average over left and right eye) in two populations of sea trout (Ii, *N *=* *90 and Ingarskila, *N *=* *82) and two populations of Atlantic salmon (Neva, *N *=* *71 and Tornio, *N *=* *58). Data overlap is visualized by the shade of the points with darker points indicating a higher overlap rate. Fitted lines show linear regressions through the origin (*R* ^2^ = .901, .916, .938, and .857, respectively). Slopes were used as measures of inverse tolerance for each fish population with lower slopes indicating less damage per parasite capita

All infected fish had cataracts and cataract coverage varied between 10% and 60% of the lens area per eye. While the interaction between parasite load and population on cataract coverage was not significant, the interaction between parasite load and species tended to be significant, suggesting that species tended to differ in cataract coverage at a given parasite load (Table 3). More specifically, the average slope of this interaction was lower for salmon than for trout, suggesting higher tolerance in salmon (Figure 4).

**Table 3:** Generalized linear model analyses of genetic variation in tolerance

Factors	*df*	χ^2^	*p*
Parasite load	1	98.6	<.001
Parasite load^2^	1	21.9	<.001
Species	2	1,595.5	<.001
Population (species)	2	0.37	.831
Parasite load × species	1	3.2	.072
Parasite load × population (species)	2	1.2	.541
Parasite load^2^ × species	1	2.8	.094
Parasite load^2^ × population (species)	2	3.9	.141

Cataract coverage is used as a measure of damage inflicted and entered as dependent variable.

On the individual level, there was a significant difference between the fish species in residual cataract coverage (GLM, *N *=* *301, $\chi_{1}^{2}$ = 24.48, *p *<* *.001) with salmon showing on average smaller residuals (−1.37 ± 0.37) than trout (1.03 ± 0.32), and thus higher tolerance. Populations, nested within species, did not differ in this measure of tolerance (Ingarskila: 0.04 ± 0.03 vs. Ii 0.11 ± 0.04 and Neva: −0.08 ± 0.03 vs. Tornio −0.11 ± 0.04; $\chi_{2}^{2}$ = 1.72, *p *=* *.424).

### 3.4 Relationship between the defense traits

**Figure 5:**
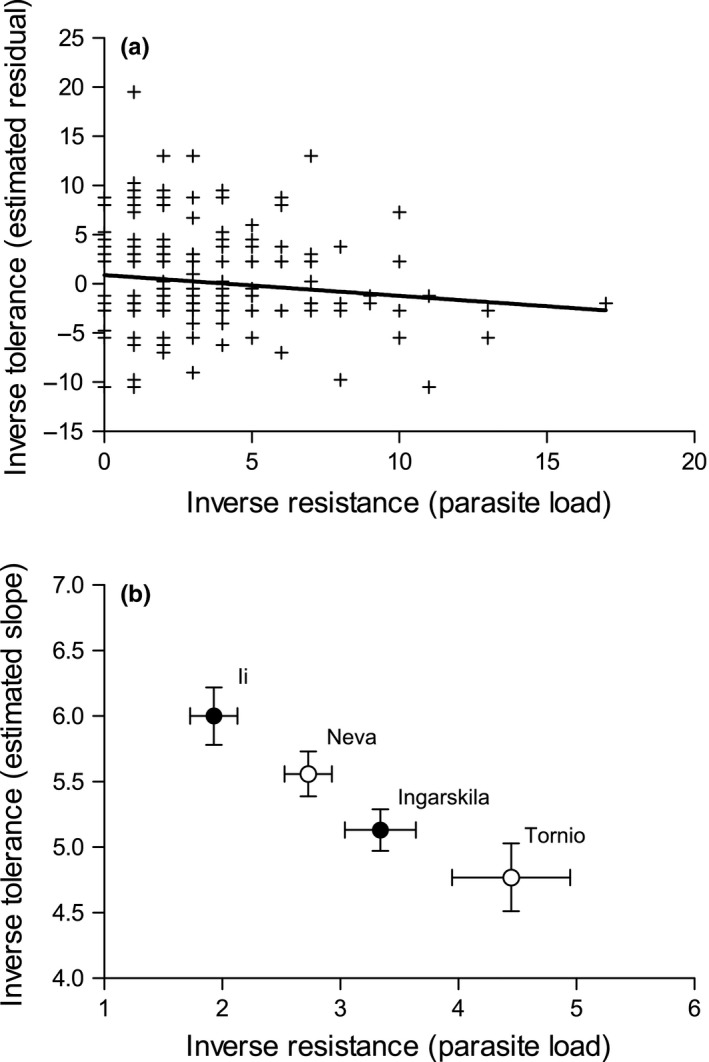
Relationship between resistance, measured inversely as parasite load after the experimental exposure, and tolerance in trout and salmon on (a) the individual level and (b) the population level. For (a), tolerance was estimated as residual deviations from a common regression line between cataract coverage and parasite load across all fish individuals, with negative values representing relatively small cataracts for a given load and thus higher tolerance. For (b), tolerance was quantified as the slope of regression of cataract coverage against parasite load across populations (see Figure 4) with lower slopes indicating less damage per parasite capita and thus higher tolerance. Closed circles represent populations of sea trout and open circles populations of Atlantic salmon. Error bars indicate standard errors

On the individual level, we found no significant relationship between avoidance behavior and resistance (only trout, *N *=* *62, *F*_2,1_ = 0.01, *p *=* *.987), or between avoidance behavior and tolerance (only trout, *N *=* *57, *F*_2,1_ = 0.21, *p *=* *.836). In contrast, resistance and tolerance were negatively related (both species, *N *=* *236, *F*_4,231_ = 2.83, *p *=* *.026), so that more resistant individuals had larger relative cataracts and thus were less tolerant to infection (Figure 5a). On the population level, there was also a strong negative correlation between tolerance and resistance (*N *=* *4, *r*_s_ = −1.000, Figure 5b), suggesting that the most resistant populations had the highest cataract coverage for a given parasite load.

## 4 Discussion

Hosts have evolved a range of adaptations that mitigate the negative impact of parasitic infections, but typically, natural host populations show also tremendous genetic variation in the defense traits. Understanding such variation is essential when considering the evolution of host defense strategies. To our knowledge, the present study is the first to simultaneously assess three different defense traits in the same host–parasite system that act in preventing parasite exposure (avoidance), reducing parasite establishment (resistance), and reducing the deleterious effects of infection (tolerance). Using two salmonid fish species and four genetically distinct populations, we demonstrate species‐specific variation in resistance and tolerance as well as population‐specific variation in resistance. Further, our results provide evidence for a trade‐off between resistance and tolerance, suggesting that the overall defense of vertebrate hosts may be a compromise between different interacting traits.

Detecting the threat of infection and avoiding or minimizing parasite exposure generally represents the first line of host defense. However, very few empirical studies have compared avoidance behavior among species and populations (but see Sears et al., 2015; Tranter, LeFevre, Evison, & Hughes, 2015), which is why our understanding of the magnitude of underlying genetic variation is limited. Here, trout clearly avoided areas containing parasites and this pattern was similar in the two genetically distinct populations. Overall, this is consistent with the results of earlier behavioral studies on rainbow trout and *D. pseudospathaceum* (Karvonen, Seppälä, & Valtonen, 2004b; Mikheev et al., 2013). Salmon, on the other hand, preferred certain areas of the experimental tank irrespective of parasite occurrence. Interestingly, parasite load acquired during the trials did not differ between the fish species, even if the load increased with the time spent in the parasite compartment in both species. Given the parasites’ passive nature of host finding primarily via vertical movements in the water body (Haas et al., 2002) and the tendency to infect within a narrow host home range (Karvonen, Paukku, Valtonen, & Hudson, 2003), it is possible that the generally lower activity of salmon compared with trout reduced the rate of parasite encounter irrespective of the preferred compartment. Consequently, this may also have minimized infection and made active avoidance less important.

Parasite recognition in this system likely works through mechanical stimuli delivered by penetrating cercariae (Karvonen et al., 2004b; Poulin, Marcogliese, & McLaughlin, 1999). This means that avoidance behavior may only be triggered after an initial parasite contact and invasion, thereafter requiring activation of immunological resistance. We also found significant variation in this trait between the fish species and populations. While the exact mechanisms underlying resistance variation are currently unclear, it can be related, for example, to the level of parasite exposure these populations have experienced in the wild. Indeed, variation in parasite pressure, mediated by host life histories or environmental components, has been shown to affect the evolution of resistance to *D. pseudospathaceum* (Kalbe & Kurtz, 2006; Lenz, Eizaguirre, Rotter, Kalbe, & Milinski, 2013; Scharsack & Kalbe, 2014; Scharsack, Kalbe, Harrod, & Rauch, 2007). Atlantic salmon and sea trout share many life‐history tactics (Klemetsen et al., 2003), but accurate homing, that is return to natal sites after sea migration, generally results in strong local adaptations (reviewed in Primmer, 2011). Thus, local parasite abundance or other environmental elements may affect the cost–benefit ratio of parasite defense and lead to genetically controlled variation.

Resistance of fish was also higher after the avoidance trials, which may reflect elevated immune activity due to this preceding exposure. Importantly, however, individual resistance was positively related among all infection events, suggesting consistency in intrinsic individual resistance regardless of the pattern of exposure and history of infection. This may be related, for example, to genetic predisposition to infection or to host condition and suggests that infections taking place in different environments during the study did not significantly influence the results.

When avoidance and resistance are incomplete, tolerance represents the third line of host defense. In this particular system, the host can only tolerate the infection once the parasite has established in the eye lens. Theoretical models of tolerance evolution predict fixation of tolerance alleles due to its positive evolutionary feedback: a reduction of parasite‐induced damages without limiting the infection increases parasite prevalence, which in turn sustains selection for tolerance (Miller, White, & Boots, 2005; Roy & Kirchner, 2000). However, experimental studies, including the present, demonstrate significant genetic variation in animal tolerance (Adelman et al., 2013; Blanchet, Rey, & Loot, 2010; Maze‐Guilmo, Loot, Paez, Lefevre, & Blanchet, 2014; Parker, Garcia, & Gerardo, 2014; Råberg et al., 2007). A theoretical study tackled the discrepancy of fixation vs. variation in tolerance, and showed that a trade‐off between resistance and tolerance, depending on the overall costs of defense, can explain variation in tolerance among natural populations (Best, White, & Boots, 2008). Such a trade‐off was also evident in our study as a lower level of resistance on the individual level was associated with lower cataract coverage per parasite capita, suggesting higher tolerance in the least‐resistant individuals. This relationship was also remarkably strong and consistent across different fish populations originating from distant geographic locations, suggesting generality of this finding. To our knowledge, this is the first study demonstrating such a trade‐off in a natural animal system (see Råberg et al., 2007 for an example in a mice‐rodent malaria system), while other studies have not detected a relationship between resistance and tolerance (Hayward et al., 2014; Lefevre, Williams, & de Roode, 2011; Maze‐Guilmo et al., 2014).

We did not find evidence for a relationship between avoidance behavior and either resistance or tolerance, which is in contrast with previous studies. For example, humans downregulate immunity during the luteal phase of the menstrual cycle to prevent attacks on a potential blastocyst, which causes an increased disgust response to avoid pathogens (Fleischman & Fessler, 2011). The latter result suggests a trade‐off between avoidance and resistance and also that avoidance behavior, while considered cost‐effective compared to other defense traits (Curtis, 2014), can show variation depending on interactions with other defense traits. However, in the present system, it is possible that avoidance is actually so cost‐effective that it makes the detection of resource trade‐offs with other defense mechanisms difficult. Moreover, as parasite avoidance in this system is triggered by the infection and thus rarely provides complete protection, investment into resistance and/or tolerance is necessary anyhow. More research is needed on possible trade‐offs between avoidance behavior and other defense mechanisms, particularly in systems where avoidance is either more effective or more expensive.

In summary, this study showed variation in different defense mechanisms in two closely related salmonid fish species, which may be related to environmental conditions such as variation in parasite pressure. We also found evidence that at least part of this variation can be explained by trade‐offs between the defense traits. As different defense mechanisms can lead to different evolutionary interactions between hosts and parasites, heterogeneities in defense traits among host populations may have profound consequences for host–parasite evolution. For example, it has been suggested that different defense strategies among hosts may have significant implications for the evolution of parasite virulence (Gandon & Michalakis, 2000; Miller, White, & Boots, 2006). Overall, this emphasizes the importance of considering all defense system components when making predictions on the outcome of host–parasite interactions.

## Conflict of Interest

None declared.
