# Supplementary material for: Vertebrate defense against parasites: Interactions between avoidance, resistance, and tolerance
Source: Ecol Evol. 2016 Dec 20;7(2):561–71. doi: 10.1002/ece3.2645 (PMC5243791; doi:10.1002/ece3.2645)
Supplement: Supplementary file 1 [file ECE3-7-561-s001.pdf]

Supplementary material

Vertebrate defence against parasites: interactions between avoidance, resistance and tolerance

Ines Klemme & Anssi Karvonen

Figure S1

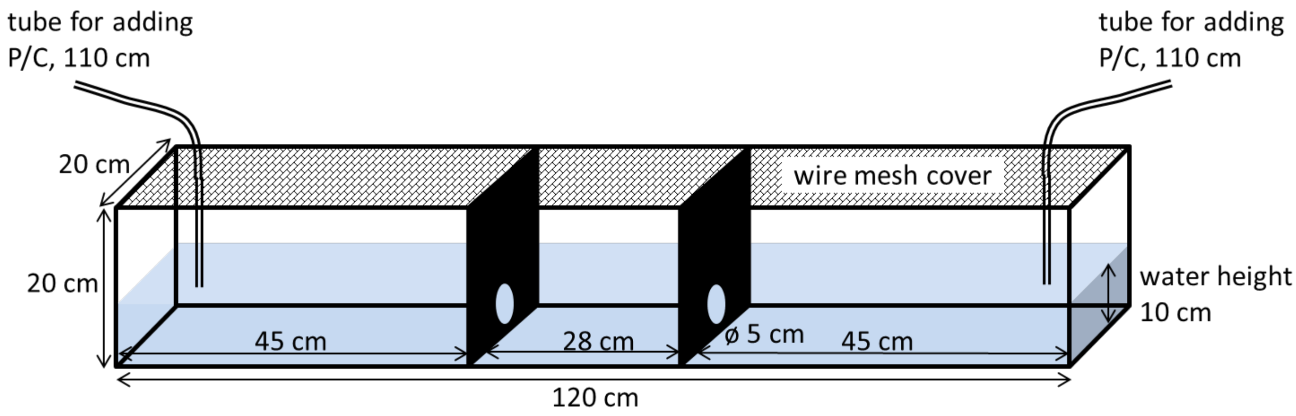

Fig. S1 Sketch of experimental tank used for avoidance behaviour tests.
